# An Empirical Model for Describing the Small Field Penumbra in Radiation Therapy

**DOI:** 10.1155/2019/7584743

**Published:** 2019-12-07

**Authors:** Shi-Qiang Tang, Yee-Min Jen, Jia-Ming Wu

**Affiliations:** ^1^Radiotherapy Center, Chenzhou No. 1 People's Hospital, Chenzhou, Hunan, China; ^2^Department of Radiation Oncology, Yee Zen General Hospital, Tao Yuan City, Taiwan; ^3^Department of Medical Physics, Chengde Medical University, Chengde City, Hebei Province, China; ^4^Department of Biomedicine Engineering, Chengde Medical University, Chengde City, Hebei Province, China

## Abstract

**Purpose:**

We developed a mathematic empirical model for describing the small field penumbra in order to analyze the potential dose perturbation caused by overlapping field to avoid the dose calculation errors in linear accelerator-based radiosurgery.

**Materials and methods:**

A ball phantom was fabricated for measuring penumbra at 4 different gantry angles in the coplanar plane. A least square root estimation (LSRE) Model was created to fit the measured penumbra dose profile and to predict the penumbra dose profile at any gantry angles. The Sum of Squared Errors (SSE) was used for finding the parameters *n* and *t* for the best fitting of the LSRE model. Geometric and mathematical methods were used to derive the model parameters.

**Results:**

The results showed that the larger the gantry angle of the field, the more the expansion of the penumbra dose profile. The least square root estimation model for describing small field penumbra is as follows: ${\text{Penumbra}}_{D\left(\text{š}\right)}=T\cdot \left(1/2\right)\cdot \left(1-\left(\text{š}/\sqrt{n+{\text{š}}^{2}}\right)\right)+t$ where Penumbra_*D*(š)_ denotes the dose profile *D*(*š*) at the penumbra region, *T* is the penumbra height (usually in scalar 100), *n* is the parameter for curvature, *š* = *x* − *W*_*d*_/2 (*x* and *š* are the values in cm on *x*-axis), and *t* is the radiation transmission of the collimator. Geometric analysis establishes the correlation between the penetration depth of the exposure and its effect on the penumbra region in ball phantom. The penumbra caused by an exposure at any arbitrary angles can be geometrically derived by using a one-variable quadratic equation.

**Conclusion:**

The dose distribution in penumbra region of small field can be created by the LSRE model and the potential overdosage or underdosage owing to overlapping field perturbation can be estimated.

## 1. Introduction

With the adoption of advanced technologies in modern radiotherapy such as stereotactic radiosurgery, stereotactic body radiation therapy, and intensity-modulated radiation therapy, there is an increased interest in the small-field dosimetry of photon beams. A beamlet used in linear accelerator-based radiosurgery is the smallest field formed by one single leaf of the MLC facing the opposite one and is only a portion of the target of dose delivery [1]. All these small fields are delivered and superimposed at different gantry angles during linear accelerator-based radiosurgery.

The analysis of small field penumbra is important in linear accelerator-based radiosurgery. It includes geometric, transmission, and photon scattered components. Geometric penumbra originates from the radiation source when it is not a single point. Transmission penumbra occurs when the beam passes through the edge of the jaw or MLC before it reaches the full attenuation point of the jaw and the MLC. Scattered radiation from the former two components is added to form the total penumbra, namely, the physical penumbra. The physical penumbra width is defined as the lateral distance from the central axis between 20% and 80% of the central axis dose at a reference depth. The height of a penumbra is defined as the intersection point of the central axis with the dose profile and is usually normalized to be 100%.

We design a mathematical model to fit the penumbra in small field used in linear accelerator-based radiosurgery. The penumbra perturbation was also investigated by our model when one field is overlapped by another segment created by MLC. The dose perturbation at the field edges could lead to a potential monitor unit calculation error; therefore, this penumbra perturbation has to be accounted for in order to obtain the correct dose modification especially in the situation of split field [2].

## 2. Materials and Methods

### 2.1. Experiment Design and Steps

The experiment was conducted in the following steps:

1. Establishment of a calibration curve for future dose quantification of irradiated films
2. Single exposures of the standard reference field and with double exposure at four different gantry angles
3. Establishment of a mathematical model, the least square root estimation (LSRE) model, and using the LSRE model to fit the penumbra dose profiles obtained in the previous step
4. Using the Sum of Square Errors (SSE) to find the values of the LSRE model parameters
5. Geometric derivation of the LSRE penumbra parameters at any gantry angle by using a transformation equation
6. Estimating the dose curve expansion owing to overlapping field perturbation in split field by using LSRE

The details of each step are described in the following sections.

### 2.2. Establishment of a Calibration Curve

We used GAF Chromic EBT2 films (ISP Technology Inc., Wayne, NJ, USA; Lot #F05090901) for the experiment. The film has a *Z*_eff_ tissue equivalence of 6.98 with a fast polymerization in the image-forming process. Its absorption peaks are independent of radiation energy. Its higher sensitivity in the dose range of 2–800 cGy and an inhomogeneity smaller than 2% after radiation exposure make it suitable for dosimetry analysis. The film processing and dose profile measurements followed the international protocols [3]. A preexposure technique was used for the calibration curve derivation [4]. This was performed by giving each film a priming dose of 2 Gy to homogenize the film density using an Elekta 6 MV linear accelerator (Stockholm, Sweden) with a dose rate of 300 MU/min. We measured the dose homogeneity using a densitometer. The previously exposed films were embedded into a solid water phantom at a depth of 5 cm. Radiation was delivered with the same 6 MV linear accelerator with a 10 × 10 cm^2^ field size at an SAD of 100 cm. Graded doses of 5, 10, 15, 40, 60, 80, 100, 150, and 200 cGy were given to obtain the Hurter-Driffield calibration curve (H-D curve).

All exposed films were then scanned with an Epson Expression 10000XL scanner in the 48-bit RGB mode (16 bits per color), and the data were saved as tagged image file format (TIFF) and analyzed by the VariSoft imaging procession software. The films were scanned in the landscape orientation to reduce the optical density (OD) variation to ≤2% [5]. The experiments and film analysis were performed at room temperature to reduce the temperature-dependent evolution of the films [6]. All OD measurements were performed 24 h after irradiation to avoid time effect on the films. GAF chromic film was handled with forceps to avoid scratching and fingerprints.

The OD of the film was derived by the following equation:

(1) $$\begin{array}{c} \text{OD}=\;\log_{10}\frac{B_{0}}{B}, \end{array}$$

where *B*_0_ is the background density, namely, the scanner signal of the unexposed film, and *B* is the scanner signal of the exposed film.

A red filter was placed on top of the GAF films before scanning to increase the slope of the H-D curve, thereby raising the resolution of the dose-OD curves [7].

### 2.3. Single Exposures of the Standard Reference Field and with Double Exposure at Four Different Gantry Angles

The single exposure of a radiation field 4 × 2 cm^2^ on dimension 10 × 10 cm^2^ GAF film was conducted in a regular 30 × 30 cm^2^ polystyrene solid phantom at the depth of 5 cm with 100 cGy as the standard reference of penumbra profile. The other four films with dimension 10 × 10 cm^2^ were exposed each with a radiation field 4 × 2 cm^2^ on GAF film in a regular 30 × 30 cm^2^ polystyrene solid phantom at the depth of 5 cm with 100 cGy as the first exposure and then follows the 6 × 4 cm^2^ field at four different gantry angles in ball phantom separately. We chose a 4 × 2 cm^2^ field for two reasons: firstly, to simulate the commonly used calculation grid size in the treatment planning systems; secondly, to mark dose profile measurement direction for a smaller filed size used in linear accelerator-based radiosurgery. The penumbra dose profile produced by this single exposure was used as the original standard reference data.

**Figure 1:**
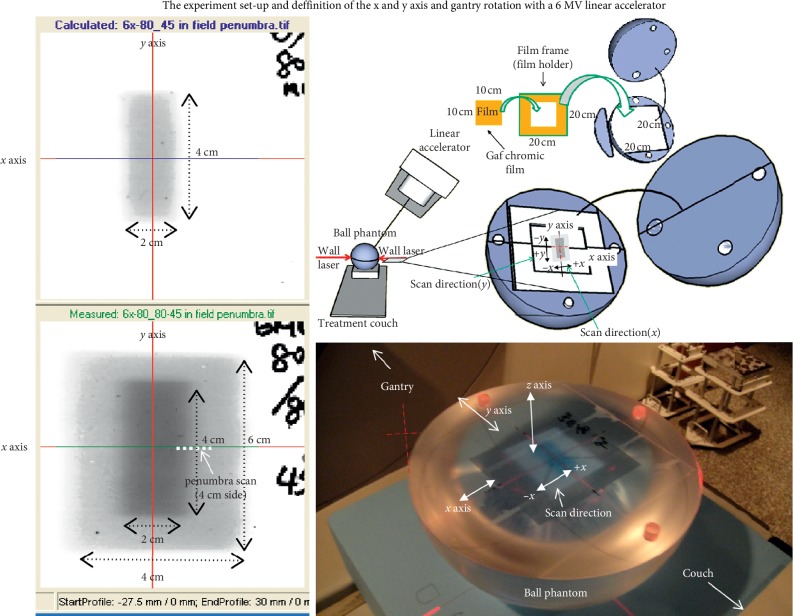
The custom-made ball phantom for the second film exposure. The *x*, *y*, and *z* axes of the film are defined on this figure. The second exposure with a 6 × 4 cm^2^ field was given to the film that was previously irradiated with a 4 × 2 cm^2^ field. The film was embedded in the equator plane with the longitudinal direction parallel to the *y* axis at different gantry angles for in-field penumbra perturbation study. Coplanar irradiation denotes that all the irradiations were delivered along the *xz* axis with the same isocenter.

An acrylic ball phantom 22 cm in diameter was made for the second exposure. A wall laser was aimed at the ball equator plane to set the SAD at 100 cm isocentrically for the second exposure. The ball could be separated from its equator into two hemispheres. One of the two hemispheres has a 20 × 20 × 0.1 cm^3^ dent on the equator plane to hold the film (Figure 1). The four previously exposed 4 × 2 cm^2^ films were put separately into the film holder on the equator plane for the second exposure with a 6 × 4 cm^2^ field at 30°, 45°, 60°, and 90° gantry angles, respectively (Figure 1).

The penetration depth remains the same at the 2 cm side on the −*y* and +*y* axis of the 4 × 2 cm^2^ field at different gantry angles (Figure 1). On the other hand, the radiation penetration depth changes at the 4 cm side of the 4 × 2 cm^2^ field on the axis of −*x* and +*x* from the larger second field. Because the gantry rotated around the *y* axis on the *xz* plane, the penetration depth from the larger second field changes only at the 4 cm side of the 4 × 2 cm^2^ field on the axis of −*x* and +*x* (Figure 1). The penetration depth at the 2 cm side remains the same. This phenomenon led to different dose depositions on the penumbra region along the 4 cm side on the −*x* and +*x* axis compared to the 2 cm side on the −*y* and +*y* axis. We therefore analyzed only the 4 cm side.

### 2.4. Establishment of a Mathematical Model to Fit the Dose Profile of the 4 × 2 cm^2^ Field after Superimposition by a 6 × 4 cm^2^ Field at Different Gantry Angles in Coplanar Plane

A mathematical model, named the least square root estimation model (LSRE model), was developed for modeling the penumbra dose profile perturbation. The goal was to build a mathematical model which could predict the penumbra profiles at any gantry angles in coplanar irradiation. The LSRE model originated from the proportion function *y*(*x*)=1/*x* . For details, please refer to Appendix A.

The least square root estimation model is expressed as follows:

(2) $$\begin{array}{c} {\text{Penumbra}}_{D\left(\text{š}\right)}=T\cdot \frac{1}{2}\cdot \left(1-\frac{\text{š}}{\sqrt{n+{\text{š}}^{2}}}\right)+t, \end{array}$$

where Penumbra_*D*(š)_ denotes the dose profile *D*(š) at the penumbra region; *T* is the penumbra height representing the intersection of the beam central axis with the penumbra curve and is between 100 and 99.8%; *n* is the parameter which gives the curve the curvature, and the greater the *n* is, the flatter the penumbra would be; *š* = *x* − *W*_*d*_/2, where *š* and  *x* are numbers on the *x* axis in the unit of cm, *W*_*d*_/2 is the half-field width or the width from the central axis to the 50% dose point of the penumbra region; and *t* is the radiation transmission of the collimator.

The LSRE model was used to fit the penumbra of the single exposure standard reference curve and the 4 × 2 cm^2^ dose profile on the 4 cm side after 6 × 4 cm^2^ field double exposures at 4 different gantry angles in a coplanar irradiation. It fits the penumbra (Penumbra_*D*(š)_) dose profile from the central axis to the end of the curve.

### 2.5. Using the Sum of Square Errors (SSE) to Find the Optimal *n* and *t* of the LSRE Model

We need a tool to determine whether a best fit to the penumbra profile had been achieved or not by the LSRE. The Sum of Square Errors (SSE) provides an ideal measurement of the differences between the measured and calculated data by the LSRE model [8]. The lowest SSE indicates a minimum difference between the LSRE model prediction and the measured data.

The SSE is defined as follows:

(3) $$\begin{array}{c} \text{SSE}=\overset{n}{\underset{i=1}{\sum }}{\left(x_{i}-\bar{x_{l}}\;\right)}^{2}, \end{array}$$

where {*x*_*i*_} is the observation and $\left\{\bar{x_{l}}\right\}$ is the means of predicted values.

The total SSE is

(4) $$\begin{array}{c} {\text{SSE}}_{\text{total}}={\text{SSE}}_{1}+{\text{SSE}}_{2}+{\text{SSE}}_{3}+{\text{SSE}}_{4}+\cdots +{\text{SSE}}_{n}. \end{array}$$

The *n* that leads to the lowest SSE would result in the best fit of the penumbra curvature; the *t* with the lowest SSE is derived separately and it affects the height of the penumbra tail.

### 2.6. Geometric Derivation of the Perturbation Effect on the Penumbra by a 6 × 4 cm^2^ Second Field at Arbitrary Angles in Coplanar Irradiation

**Figure 2:**
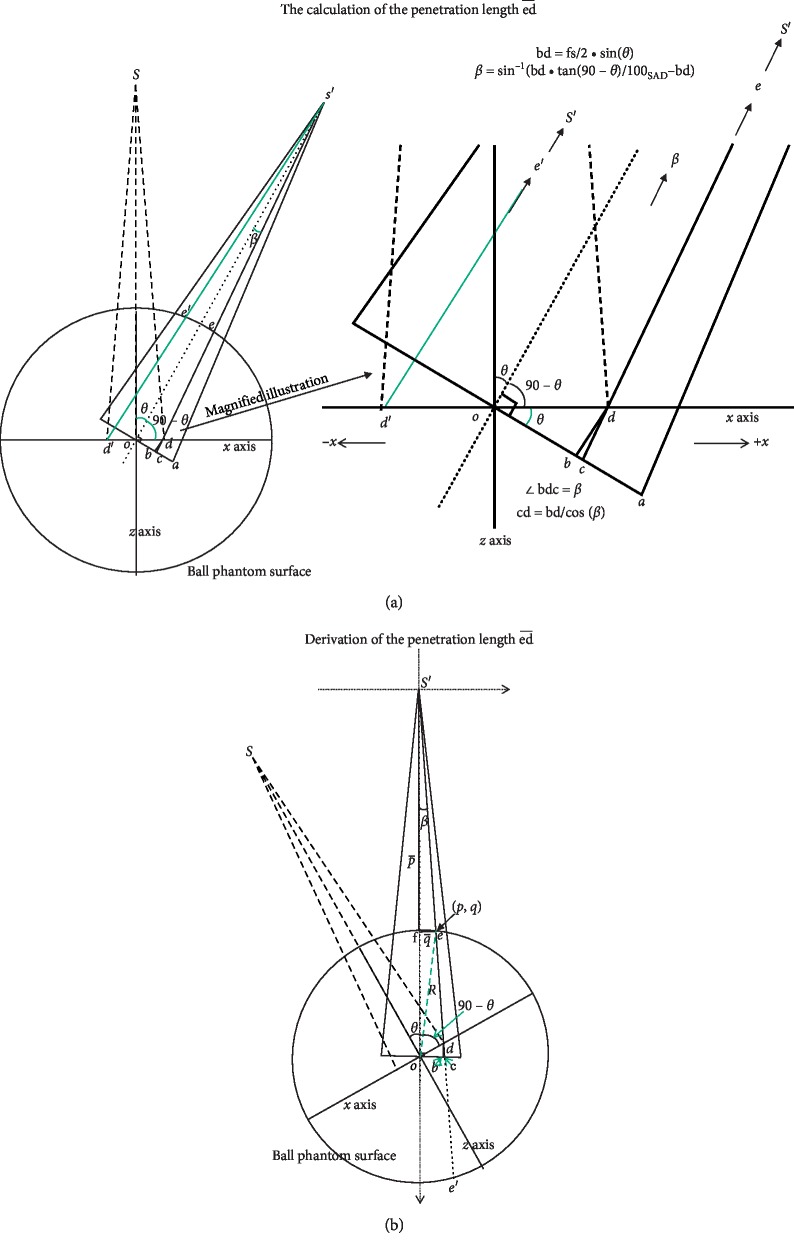
(a) The calculation of the length $\bar{\text{ed}}$ at a gantry angle of *θ* on $\bar{{\text{s}}^{\prime }\text{c}}$ by geometric method after double exposures with a larger and a smaller field. The larger field was 6 × 4 cm^2^ and the smaller field was 4 × 2 cm^2^ field, respectively. *S* represents the linear accelerator radiation source position with a gantry angle of 0°, and S′ denotes the source position after a gantry rotation of *θ* degrees. *o* is the rotational isocenter; *d* is the field edge of the small field delivered at 0° at the 4 cm side of the 4 × 2 cm^2^ field; *a* indicates the field edge of the larger field at the 6 cm side of the 6 × 4 cm^2^ field when the gantry rotates to the angle of *θ*. $\bar{\text{od}}$ is the half-field size of the smaller field on the 4 cm side of the 4 × 2 cm^2^ field while $\bar{\text{oa}}$ is the half-field size of the larger field on the 6 cm side of the 6 × 4 cm^2^ field. fs/2 denotes the half-field size on the 4 cm side of the 4 × 2 cm^2^ field ($\bar{\text{od}}$) and FS/2 denotes the half-field size on the 6 cm side of the 6 × 4 cm^2^ field (FS/2). (b) This figure shows the geometric derivation of $\bar{\text{ed}}$. $\bar{\text{ed }}$ is the key factor controlling the dose distribution from the larger field onto the penumbra region of the smaller field along $\;\bar{\text{od}.}$ $\;\bar{\text{ed}}$ = $\bar{{\text{S}}^{\prime }\text{c}}$ − $\bar{\text{cd}}$ − $\bar{{\text{S}}^{\prime }\text{e}}$, where $\bar{{\text{S}}^{\prime }\text{c}}=100/\cos\left(\beta \right)$ , and $\bar{\text{cd}}$ = $\bar{\text{bd}}/\text{cos}\left(\beta \right)$. For details, please refer to Appendix B.

So far, we have the best fits of parameters *n* and *t* only for the dose profiles at gantry angles of 30°, 45°, 60°, and 90°. Eventually, we aim to obtain *ns* and *ts* at any gantry angles. For this, we need to find the lengths of $\bar{\text{ed}}$ at a specific gantry angle because the parameters *n* and *t* were dependent only on the penetration depth $\bar{\text{ed}}$. $\bar{\text{ed}}$ can be derived by using geometry (Figure 2(a)). $\bar{\text{ed}}$ is the penetration length on the $\bar{\text{S}\prime \text{c}}$ of the larger field at a gantry angle of *θ*. It is the distance where the radiation traverses inside the phantom before intersecting with the penumbra edge of the previous field. It is the only factor controlling the dose contribution from the larger superimposing field onto the smaller field along $\;\bar{\text{od}}$. The larger $\bar{\text{ed}}\;$ is, the more the attenuation of the passing radiation will be with less contribution to the penumbra. $\bar{\text{ed}}$ is also the penetration depth on the $\bar{\text{S}\prime \text{c}}$ of the 6 × 4 cm^2^ field. $\bar{\text{S}\prime \text{c}}$ is the radiation line within the penumbra region from the source S′ in the larger field along the +*x* and −*x* at an arbitrary gantry angle of  *θ*. For the detailed geometric derivation of $\bar{\text{ed}}$, please refer to Figure 2(b) and Appendix B.

## 3. Results

### 3.1. Calibration Curve

**Figure 3:**
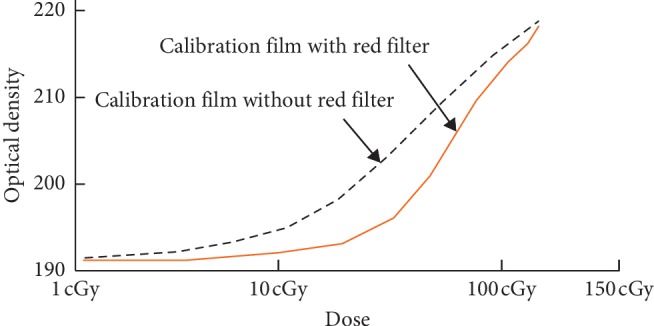
The calibration curve was steeper and thus more sensitive when the film was scanned with a red color filter.

When the film was scanned with a red color filter, the calibration curve was steeper and thus with a higher OD-dose resolution. Radiation dose was then derived by the OD method (please refer to Figure 3).

### 3.2. The Dose Profile of the 4 × 2 cm^2^ Field with a Second Exposure by a 6 × 4 cm^2^ Field at 4 Different Gantry Angles in Coplanar Irradiation

**Figure 4:**
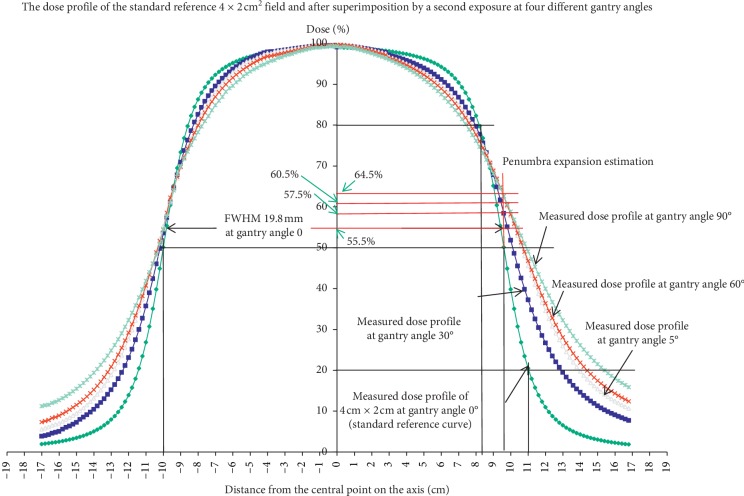
The dose profile of the smaller 4 × 2 cm^2^ field irradiated at a gantry angle of 0° at the flat polystyrene solid phantom, followed by a second exposure in the ball phantom with a larger field of 6 × 4 cm^2^ at different gantry angles. The dose profile changes after second exposures at a gantry angle different from the first exposure. It also shows the full-width half maximum (the width at 50% dose of the −*x* axis and the +*x* axis) and the penumbra width from 80% to 20% of the central axis.

Figure 4 demonstrates the single exposure as the standard reference penumbra profile and the cumulative dose profiles after double exposures on 4 × 2 cm^2^ with a 6 × 4 cm^2^ field at 4 different gantry angles. The dose profiles on the + axis are slightly higher than the dose profile on the −*x* side. This is caused by smaller penetration depths $\bar{\text{ed}}\text{s}$ on the +*x* axis than $\bar{{\text{e}}^{\prime }{\text{d}}^{\prime }}\text{s}$ at the −*x* side in the ball phantom (Figure 2(a)). The Full-Width Half Maxima (FWHM, the width between 50% dose of the −*x* axis and the +*x* axis) was 19.8 mm after single irradiation at a gantry angle of 0 in the flat polystyrene solid phantom. The FWHM became 20.3 mm, 21.1 mm, 21.3 mm, and 21.6 mm after second exposures at gantry angles of 30°, 45°, 60°, and 90° in the ball phantom, respectively (Figure 4). The penumbra width between 80% to 20% of the central axis was 2.8 mm after a single exposure at 0° with a 4 × 2 cm^2^ field (Figure 4). After the second exposure, they were 4.7 mm, 6.2 mm, 6.7 mm, and 8.1 mm at gantry angles of 30°, 45°, 60°, and 90°, respectively (Figure 4). The renormalized dose profile shows, as expected, no change of the penumbra width after double exposures of any doses with a 6 × 4 cm^2^ field as long as the exposures were delivered at the same gantry angle as the first. The dose profile changes only after double exposures delivered at different gantry angles.

### 3.3. Dose Profile Fitting Using the LSRE Model

**Figure 5:**
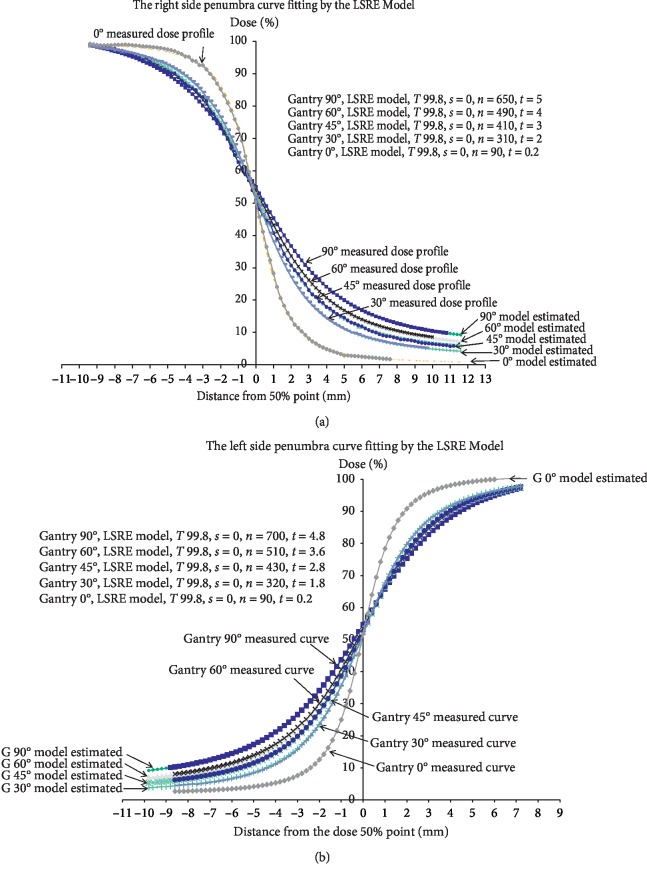
(a) The right-side penumbra dose profiles vary with the gantry angles of the second exposure. The curvature of the right-side penumbra region can be fitted using the LSRE model by adjusting the (*n*) *T* and (*t*). (b) The left-side penumbra dose profiles vary with the gantry angles of the second exposure. The curvature of the left-side penumbra region can be fitted using the LSRE model by adjusting the (*n*) *T* and (*t*). The “90° measured curve” and “90° model estimated” curve denote the dose profile measured at the gantry angle of 90° in the ball phantom and the predicted curve at the same. They overlap each other completely.

Parameters *T*, *n*, and *t* in the LSRE model determine the dose profile shape (equation (2)). The right-side dose profile after the first exposure at a gantry angle of 0° can be fitted accurately with *T* = 99.8, *n* = 90, and *t* = 0.2 (Figure 5(a)). *n* and *t* that result in the best fit, denoted as (*n*, *t*), at gantry angles of 30°, 45°, 60°, and 90°, are (310, 2), (410, 3), (490, 4), and (650, 5), respectively.

The measured curves of the left-side dose region can also be estimated by adjusting the *n*, T, and *t* (Figure 5(b)). Parameters *n* and *t* of the left-side curve at 0 gantry angle after single exposure were (90, 0.2). After the second exposures, the parameters (*n*, *t*) were (320, 1.8), (430, 2.8), (510, 3.6), and (700, 4.8) at gantry angles of 30°, 45°, 60°, and 90°, respectively.

### 3.4. Sum of Square Errors (SSE): Finding the Best *n* and *t*

**Figure 6:**
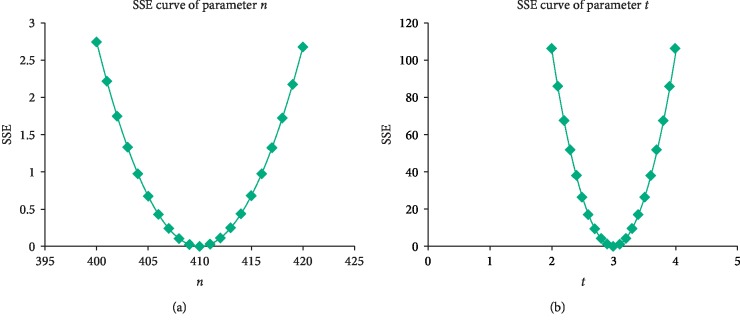
(a, b) These figures demonstrate the use of the SSE to find the best *n* and *t* of the LSRE model at the gantry angle of 45°.

Figures 6(a) and 6(b) show *n* and *t* with the smallest SSE which gives the best LSRE model fitting of the measured data at the gantry angle of 45°. The best fit at other gantry angles can be done in the same manner.

### 3.5. Geometric Derivation of the Effects on the Penumbra by Second Exposures at Arbitrary Gantry Angles

**Figure 7:**
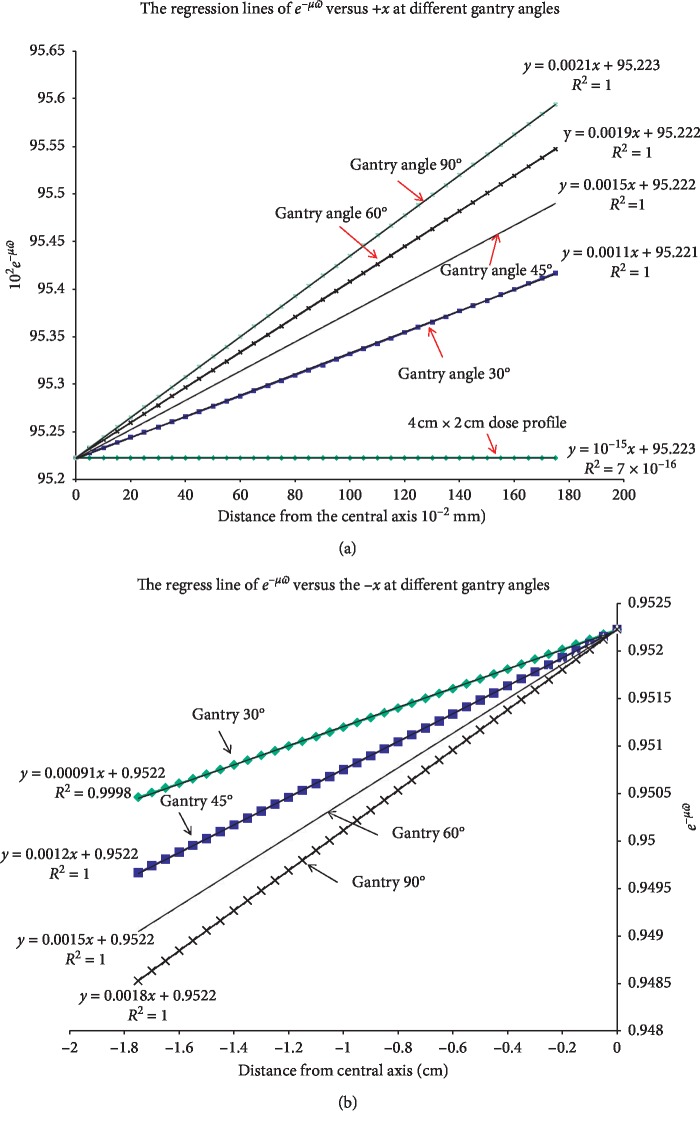
(a, b) The regression lines of e^−*μϖ*^, where *ϖ* = $\;\bar{\text{ed}}$, versus the distance on the +*x* axis in (a) and the −*x* axis in (b), at different gantry angles. The slopes are 0.0021, 0.0019, 0.0015, and 0.0011 at gantry angles of 90°, 60°, 45°, and 30°, respectively.

We found that the relationship of *e* ^−*μϖ*^ with the *x* is a straight line (Figures 7(a) and 7(b) and Table 1), where *ϖ* denotes the penetration depth $\bar{\text{ed}},\mu$ is the attenuation coefficient, and  *μ* = 0.00494 cm^−1^ of 6 MV in water. The linear regression of e^−*μϖ*^ versus *x* at different gantry angles can be described in the form of *y*=*ux*+*v*, where *u* is the slope of the regression line shown in Figures 7(a) and 7(b).  Figure 7(a) demonstrates the 4 lines with their slopes at the gantry angles of 30°, 45°, 60°, and 90°. The slopes at any other gantry angles can be derived by interpolation from these 4 know angles.

### 3.6. Derivation of the Parameters *n* and *t* of the Penumbra Dose Profiles at Arbitrary Gantry Angles Using Mathematical Methods

$\;\bar{\text{ed}}$ = $\bar{{\text{S}}^{\prime }\text{c}}$ − $\bar{\text{cd}}$ − $\bar{{\text{S}}^{\prime }\text{e}}$, where $\bar{{\text{S}}^{\prime }\text{c}}=100/\cos\left(\beta \right)$, $\bar{\text{cd}}$ = $\bar{\text{bd}}/\cos\left(\beta \right)$, and

$\bar{\text{ed}}$ is the penetration depth of the $\;\bar{{\text{S}}^{\prime }\text{c}}\;$ from the larger field in the ball phantom.$\;\bar{{\text{S}}^{\prime }\text{e}}$ can be derived from equations (5)–(7) (for details, please refer to Appendix B).

(5) $$\begin{array}{c} p^{2}-2\text{sp}+s^{2}+q^{2}=R^{2}, \end{array}$$

(6) $$\begin{array}{c} p=\frac{2s\pm \sqrt{4s^{2}-4\left(1+\xi^{2}\right)\left(s^{2}-R^{2}\right)}}{2\left(1+\xi^{2}\right)}, \end{array}$$

(7) $$\begin{array}{c} \bar{{\text{S}}^{\prime }\text{e}}=\sqrt{p^{2}+q^{2}}=\sqrt{\left(1+\xi^{2}\right)p^{2}}=\frac{s-\sqrt{s^{2}-\left(1+\xi^{2}\right)\left(s^{2}-R^{2}\right)}}{\sqrt{\left(1+\xi^{2}\right)}}. \end{array}$$

Table 1 shows the calculated $\bar{\text{ed}}\text{s}$ in Figure 2(b) in the ball phantom at different gantry angles in coplanar irradiation.

**Figure 8:**
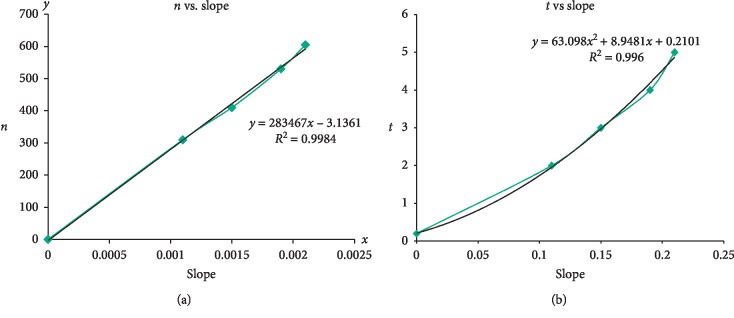
(a-b) The curves of slope versus *n* and *t* were shown in (a, b), respectively. Once the relationships of gantry angle versus slope and slope versus *n* and *t* are known, the parameters *n* and *t* in the LSRE model can be found easily to fit the measured dose profile or to construct the estimated profile for any gantry angle at coplanar irradiation with good agreement.

The regression curves for slopes versus *n* and *t* were shown in Figures 8(a) and 8(b), respectively. Therefore, once we obtain the slope of a regression curve from Figures 7(a) and 7(b), *n* and *t* can be found in Figures 8(a) and 8(b). This enables us to derive the estimated curve with precision and thus the accurate effects on the penumbra of a second irradiation at any gantry angles without the labor conducting the actual experiment.

## 4. Discussion

We developed the LSRE model for studying the penumbra perturbation incurred by field overlapping in IMRT. This model has several advantages for penumbra profile fitting. Several functions, such as cosine [9] and exponential [10] functions, have been used in commercially available planning systems to model the penumbra dose profiles of megavoltage X-ray beams. However, both functions have the disadvantage of being noncontinuous. For example, the exponential function describing the distribution of penumbra dose profile can be expressed as the edge-spread function (ESF) or the line-spread function (LSF) [11]. According to their mathematical models, the penumbra is fitted by

(8) $$\begin{array}{c} \text{ESF}\left(x\right)=Ae^{\alpha x}+Be^{\beta x}\left(x\text{ at the negative direction}\right),\\ \text{ESF}\left(x\right)=1-Ae^{\alpha x}-Be^{-\beta x}\left(x\text{ at the positive direction}\right),\\ \text{LSF}\left(x\right)=A\alpha e^{-\alpha \left|x\right|}+B\beta e^{-\beta \left|x\right|}, \end{array}$$

where *x* is the distance from the beam edge, namely, center of penumbra, and *A* + *B* = 0.5.

**Figure 9:**
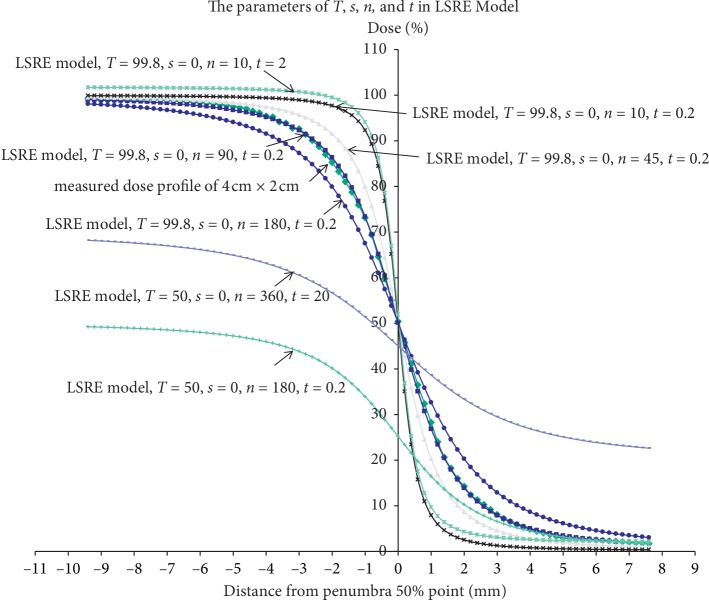
The dose profile in the penumbra region on the *x* axis can be modified by adjusting the *T s*, *t*, and *n* in the LSRE model to fit the measured dose profile.

A, B, α, and *β* are constants. The weak point of this type of penumbra function is the discrete nature in describing the penumbra curve. On the contrary, the LSRE is based on an inverse square root function [12, 13] and is a continuous equation that can fit the entire dose profile along the *x* axis (Figure 9).

Using our model, *n* and *t* can be derived easily for the measured and predicted penumbra at any gantry angles once given the gantry angle and then the slope and eventually *n* and *t*. For example, if we replace *x* with the slope from Figure 7(a) into the equation *y* = 283467*x* − 3.1361, then we get the *y* value as *n* (Figure 8(a)); if we replace *x* with the slope of a known gantry angle from Figure 7(b) into the equation *y* = 63.098*x* ^2^ + 8.9481*x* + 0.2101, then we get the *y* value as *t* (Figure 8(b)). The parameters *n* and *t* in the LSRE model can then be used to predict the dose profile with precision for any gantry angles at coplanar irradiation. This method enables radiation physicists and treatment planning system programmers to obtain penumbra dose perturbation data by using a mathematical process.

Penumbra region dose data are important in radiotherapy planning systems for monitor unit calculation especially in linear accelerator-based radiosurgery. The precise position of the MLC and the penumbra shape are two important factors for treatment monitor unit calculation. The MLC-related dose penumbra in the treatment planning system must be calibrated to avoid underdosage or overdosage.

**Figure 10:**
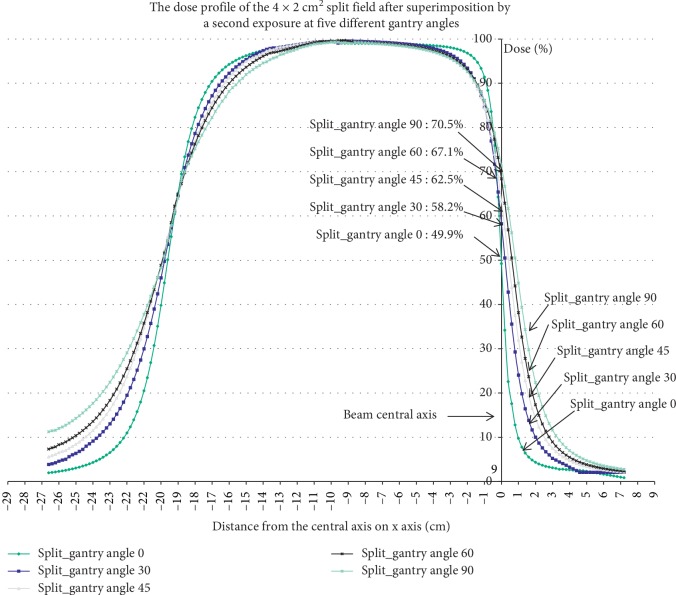
The intersection change of the dose profile perturbed by overlapping fields with beam central axis in 4 × 2 cm^2^ split field was calculated by LSRE in this study.

For example, the penumbra expansion perturbed by overlapping fields in 4 × 2 cm^2^ split field calculated in Figure 10 leads to an intersection change with the standard undisturbed 50% field edge dose profile of 70.5%, 67.1%, 62.5%, and 58.2% at 90°, 60°, 45°, and 30° gantry angles, respectively. According to this result, if we ignore tentatively the penumbra position offset correction, the split field monitor unit calculated by the escalated output could result in an overdosage up to 10.5% in linear accelerator-based radiosurgery technique [14].

Many lung cancer patients who undergo radiation therapy are treated with higher-energy photons such as 10 MV or higher to obtain a deeper penetration and better dose uniformity. However, lower energy such as 6 MV photon beams should be preferred over higher energies photons because of the significant loss of lateral dose equilibrium for high-energy beams in the low-density medium. Any gains in radial dose uniformity across steep density gradients for higher-energy beams must be weighed carefully against the lateral beam degradation due to penumbra widening. The LSRE model can be applied to predict the penumbra perturbation of both high- and low-energy photon beams in lesions surrounded by low-density organ such as lung.

## 5. Conclusion

Our study shows how to perform an accurate calculation of the penumbra perturbation caused by an overlapping field at different gantry angles by using a mathematical model in linear accelerator-based radiosurgery. The LSRE model is the first continuous equation which describes the dose profile across the entire penumbra. The clinical significance of this study is that the treatment monitor units can be overestimated or underestimated during linear accelerator-based radiosurgery if the penumbra dose profile is not correctly calculated in the treatment planning system. The LSRE model offers a mathematical approach to correct the penumbra and makes it unnecessary to do tedious physics experiments.

## Figures and Tables

**Table 1:** The coplanar penetration depth $\bar{e\;d}$(*ϖ*) and e^−*μϖ*^ on the right-side penumbra after double exposures.

Distance from central axis of point *d* on the *x* axis (cm)	Gantry angles									
	0°		30°		45°		60°		90°	
	$\bar{\text{ed}}\left(\varpi \right)$	*e* ^−*μϖ*^	$\bar{\text{ed}}\left(\varpi \right)$	*e* ^−*μϖ*^	$\bar{\text{ed}}\left(\varpi \right)$	*e* ^−*μϖ*^	$\bar{\text{ed}}\left(\varpi \right)$	*e* ^−*μϖ*^	$\bar{\text{ed}}\left(\varpi \right)$	*e* ^−*μϖ*^
0	11.00	0.95223^#^	11.00	0.95223	11.00	0.95223	11.00	0.95223	11.00	0.95223
0.1	11.00	0.95223	10.95	0.95233	10.93	0.95238	10.91	0.95241	10.90	0.95244
0.2	11.00	0.95223	10.90	0.95244	10.86	0.95253	10.83	0.95260	10.80	0.95265
0.3	11.00	0.95223	10.85	0.95255	10.79	0.95268	10.74	0.95278	10.70	0.95286
0.4	10.99	0.95223	10.80	0.95266	10.71	0.95283	10.65	0.95296	10.60	0.95308
0.5	10.99	0.95223	10.74	0.95277	10.64	0.95298	10.56	0.95315	10.50	0.95329
0.6	10.99	0.95223	10.69	0.95287	10.57	0.95313	10.48	0.95333	10.40	0.95350
0.7	10.98	0.95223	10.64	0.95298	10.50	0.95329	10.39	0.95352	10.30	0.95371
0.8	10.98	0.95223	10.58	0.95309	10.42	0.95344	10.30	0.95370	10.20	0.95393
0.9	10.97	0.95223	10.53	0.95321	10.35	0.95359	10.21	0.95389	10.10	0.95414
1	10.96	0.95223	10.47	0.95332	10.27	0.95375	10.12	0.95407	10.00	0.95435
1.1	10.96	0.95223	10.42	0.95343	10.20	0.95390	10.04	0.95426	9.90	0.95456
1.2	10.95	0.95223	10.36	0.95354	10.13	0.95406	9.95	0.95445	9.80	0.95477
1.3	10.94	0.95223	10.30	0.95365	10.05	0.95421	9.86	0.95463	9.70	0.95499
1.4	10.93	0.95223	10.25	0.95377	9.97	0.95437	9.77	0.95482	9.60	0.95520
1.5	10.92	0.95223	10.19	0.95388	9.90	0.95452	9.68	0.95501	9.50	0.95541
1.6	10.91	0.95223	10.13	0.95400	9.82	0.95468	9.59	0.95519	9.40	0.95562
1.7	10.90	0.95223	10.07	0.95411	9.75	0.95483	9.50	0.95538	9.30	0.95584

^#^Data are calculated from $\bar{\text{ed}}$ ($\bar{\text{ed}}=\varpi$) by using *e* ^−*μϖ*^, *μ* = 0.00494 cm^−1^ at 6 MV in water.

## Data Availability

The GAF chromic films and manipulation process data used to support the findings of this study were supplied by Jia-Ming Wu under license and so cannot be made freely available. Requests for access to these data should be made to Jia-Ming Wu, Ph.D., Department of Radiation Oncology, Yee Zen General Hospital, #30, Alley 321, Yuan Xin North Road, Tao Yuan, Taiwan (e-mail: jiaming.wu@chmsc.com).

## Appendix

### A. The Conceptual Process of LSRE Model

The LSRE model originated from the proportion function *y*(*x*)=1/*x*. When *x* increases from −∞ to 0, the curve of *y* is at the (−, −) quadrant. When *x* goes from 0 to +∞, the curve of *y* is situated at the (+, +) quadrant. Let 1/*x* become 1/|*x*|; then the curve falls in the (−, +) and the (+, +) quadrants. The curve of *y*(*x*)=1/|*x*| has a dose-profile-like pattern. Let $y\left(x\right)=\left(1/\left|x\right|\right)=\left(1/\sqrt{\left(x^{2}\right)}\right)$. When *x* = 0, *y* becomes infinite which does not happen in real dose profiles. Therefore, we insert *n* into *y*(*x*) to be $y\left(x\right)=\left(1/\sqrt{n+\left(x^{2}\right)}\right)$, where *n* > 0. It avoids the infinite divergence when *x* = 0. If we let $y\left(x\right)=\left(x/\sqrt{n+\left(x^{2}\right)}\right)$, then *y* approaches −1 and 1 when *x* goes from −∞ to +∞, respectively. Furthermore, in order to converge the *y* value to 1 and 0 when *x* approaches −∞ and +∞, let $y\left(\text{š}\right)=\left(1/2\right)\left(1-\left(\text{š}/\sqrt{n+{\text{š}}^{2}}\right)\right)$, where *š* = *x* − *W*_*d*_/2 (*W*_*d*_/2 is the half-field width, namely, the width from the central axis to the 50% dose point of the penumbra region). Therefore, *š* = 0 if *x*  = *W*_*d*_/2. The equation $y\left(\text{š}\right)=\left(1/2\right)\left(1-\left(\text{š}/\sqrt{n+{\text{š}}^{2}}\right)\right)$ results in *y*(*š*) as 1 and  1/2 when *x* goes from −∞ to the field edge (when *x* = *W*_*d*_/2, then *š* = 0 and *y*(š)=1/2) and *y*(*š*) as 1/2 and 0 when *x* goes from the field edge to +∞. Finally, the LSRE model can be expressed as follows:

(A.1) $$\begin{array}{c} {\text{Penumbra}}_{D\left(\text{š}\right)}=T\cdot \frac{1}{2}\cdot \left(1-\frac{\text{š}}{\sqrt{n+{\text{š}}^{2}}}\right)+t. \end{array}$$

### B. The Derivation of Radiation Attenuation Thickness in Ball Phantom

In Figure 2(a).

∠bod = *θ*, where point *b* is the intersection of the vertical line from point *d* to $\bar{\text{oa}}\;$,

(B.1) $$\begin{array}{c} \bar{\text{od}}=\frac{\text{fs}}{2},\\ \bar{\text{bd}}=\frac{\text{fs}}{2}\text{sin}\left(\theta \right),\\ \beta =\tan^{-1}\;\left(\frac{\bar{\text{bd}}\;\tan\left(90-\theta \right)}{100-\text{bd}}\right)\\ \text{or }\beta =\tan^{-1}\;\left(\frac{\bar{\text{od}}\cos\left(\theta \right)}{100}\right),\\ ∠\text{bdc}=\text{fvc,}\\ \bar{\text{cd}}\;=\frac{\bar{\text{bd}}}{\text{cos}\left(\beta \right)}\;,\\ \bar{{\text{S}}^{\prime }\text{c}}=\;\frac{100}{\text{cos}\left(\beta \right)}, \end{array}$$

and $\bar{\text{ed}}$ is the penetration depth of the $\;\bar{{\text{S}}^{\prime }\text{c}}\;$ from the larger field in the ball phantom. Because $\bar{\text{ed}}$  = $\bar{{\text{S}}^{\prime }\text{c}}$ − $\bar{\text{cd}}$ − $\bar{{\text{S}}^{\prime }\text{e}}$, we have to solve the only unknown segment $\bar{{\text{S}}^{\prime }\text{e}}$ for deriving the value of $\bar{\text{ed}}$.

Figure 2(b) is the magnification of the left portion in Figure 2(a). In Figure 2(b), point *e*_(*p*, *q*)_ is the intersection of $\bar{{\text{S}}^{\prime }\text{c}}$ with the ball surface. $\bar{{\text{S}}^{\prime }\text{e}}{\;}^{2}$ = *p* ^2^ + *q* ^2^, *p* is the distance of $\bar{{\text{S}}^{\prime }\text{f}}$, and *q* is the distance of $\bar{{\text{S}}^{\prime }\text{e}}$. Point *f* is the intersection of the horizontal line from point *e* with $\bar{{\text{S}}^{\prime }\text{o}}$; in other words, the hinge angle is 90° of $\bar{{\text{S}}^{\prime }\text{o}}$ with $\bar{\text{ef}}$.

$\bar{{\text{S}}^{\prime }\text{e}}$ is derived as follows:

(B.2) $$\begin{array}{c} q=\tan\;\beta \cdot p=\xi p\left(\xi \text{ is the slope of }\bar{{\text{S}}^{\prime }\text{e}}\right). \end{array}$$

(*s* − *p*)^2^ + *q* ^2^ = *R* ^2^ (*s* = 100 cm and is the source-axis distance which differs from the symbol s used in the LSRE model; distance *R* is the radius of the ball phantom).

(B.3) $$\begin{array}{c} ∴p^{2}-2\text{sp}+s^{2}+q^{2}=R^{2}. \end{array}$$

If we substitute *q* of equation (B.5) with equation (B.3), then

(B.4) $$\begin{array}{c} p^{2}-2\text{sp}+s^{2}+\xi^{2}p^{2}=R^{2}, \end{array}$$

(B.5) $$\begin{array}{c} p=\frac{2s\pm \sqrt{4s^{2}-4\left(1+\xi^{2}\right)\left(s^{2}-R^{2}\right)}}{2\left(1+\xi^{2}\right)}. \end{array}$$

There are two intersection points of ray line $\bar{{\text{S}}^{\prime }{\text{e}}^{\prime }}$ with the ball phantom surface which can be expressed as $2s+\sqrt{4s^{2}-4\left(1+\xi^{2}\right)\left(s^{2}-R^{2}\right)}/2\left(1+\xi^{2}\right)$ (point *e*′) and $2s-\sqrt{4s^{2}-4\left(1+\xi^{2}\right)\left(s^{2}-R^{2}\right)}/2\left(1+\xi^{2}\right)$ (point *e*) or as $2s\pm \sqrt{4s^{2}-4\left(1+\xi^{2}\right)\left(s^{2}-R^{2}\right)}/2\left(1+\xi^{2}\right)$ for the 2 points of *e* and *e*′. Point *e* is near *S*′, while point *e*′ is far from *S*′ (Figure 2(b)). We choose point *e*, namely, $\;2s-\sqrt{4s^{2}-4\left(1+\xi^{2}\right)\left(s^{2}-R^{2}\right)}\;$ for the calculation of *p*: 

(B.6) $$\begin{array}{c} ∴p=\frac{2s-\sqrt{4s^{2}-4\left(1+\xi^{2}\right)\left(s^{2}-R^{2}\right)}}{2\left(1+\xi^{2}\right)}. \end{array}$$

Since *q* = *ξp*,

(B.7) $$\begin{array}{c} ∴\bar{{\text{S}}^{\prime }\text{e}}=\sqrt{p^{2}+q^{2}}=\sqrt{\left(1+\xi^{2}\right)p^{2}}=\frac{s-\sqrt{s^{2}-\left(1+\xi^{2}\right)\left(s^{2}-R^{2}\right)}}{\sqrt{\left(1+\xi^{2}\right)}}. \end{array}$$

Once $\bar{\text{cd}},\;\bar{{\text{S}}^{\prime }\text{c}}$, and $\bar{{\text{S}}^{\prime }\text{e}}$ are derived, then $\bar{\text{ed}}$ can be obtained:

$\bar{\text{ed}}$ = $\bar{{\text{S}}^{\prime }\text{c}}$ − $\bar{\text{cd}}$ − $\bar{{\text{S}}^{\prime }\text{e}}$.
